# Postmenopausal hormones and sleep quality in the elderly: a population based study

**DOI:** 10.1186/1472-6874-10-15

**Published:** 2010-05-04

**Authors:** Gregory J Tranah, Neeta Parimi, Terri Blackwell, Sonia Ancoli-Israel, Kristine E Ensrud, Jane A Cauley, Susan Redline, Nancy Lane, Misti L Paudel, Teresa A Hillier, Kristine Yaffe, Steven R Cummings, Katie L Stone

**Affiliations:** 1 CPMC Research Institute, San Francisco, CA, USA; 2 Department of Psychiatry, University of California San Diego, San Diego, CA, USA; 3 Division of Epidemiology and Community Health, University of Minnesota, Minneapolis, MN, USA; 4 Center for Chronic Disease Outcomes Research, Minneapolis VA Medical Center, Minneapolis, MN, USA; 5 Department of Medicine, University of Minnesota, Minneapolis, MN, USA; 6 Department of Epidemiology, University of Pittsburgh, Pittsburgh, PA, USA; 7 Department of Medicine and Center for Clinical Investigation, Case Western Reserve University, Cleveland, OH, USA; 8 Department of Medicine, University of California Davis Medical School, Sacramento, CA, USA; 9 Kaiser Permanente Center for Health Research Northwest/Hawaii, Portland, OR, USA; 10 Departments of Psychiatry, Neurology, and Epidemiology, University of California, San Francisco and the San Francisco VA Medical Center, San Francisco, CA, USA

## Abstract

**Background:**

Sleep disturbance and insomnia are commonly reported by postmenopausal women. However, the relationship between hormone therapy (HT) and sleep disturbances in postmenopausal community-dwelling adults is understudied. Using data from the multicenter Study of Osteoporotic Fractures (SOF), we tested the relationship between HT and sleep-wake estimated from actigraphy.

**Methods:**

Sleep-wake was ascertained by wrist actigraphy in 3,123 women aged 84 ± 4 years (range 77-99) from the Study of Osteoporotic Fractures (SOF). This sample represents 30% of the original SOF study and 64% of participants seen at this visit. Data were collected for a mean of 4 consecutive 24-hour periods. Sleep parameters measured objectively included total sleep time, sleep efficiency (SE), sleep latency, wake after sleep onset (WASO), and nap time. All analyses were adjusted for potential confounders (age, clinic site, race, BMI, cognitive function, physical activity, depression, anxiety, education, marital status, age at menopause, alcohol use, prior hysterectomy, and medical conditions).

**Results:**

Actigraphy measurements were available for 424 current, 1,289 past, and 1,410 never users of HT. Women currently using HT had a shorter WASO time (76 vs. 82 minutes, P = 0.03) and fewer long-wake (≥ 5 minutes) episodes (6.5 vs. 7.1, P = 0.004) than never users. Past HT users had longer total sleep time than never users (413 vs. 403 minutes, P = 0.002). Women who never used HT had elevated odds of SE <70% (OR,1.37;95%CI,0.98-1.92) and significantly higher odds of WASO ≥ 90 minutes (OR,1.37;95%CI,1.02-1.83) and ≥ 8 long-wake episodes (OR,1.58;95%CI,1.18-2.12) when compared to current HT users.

**Conclusions:**

Postmenopausal women currently using HT had improved sleep quality for two out of five objective measures: shorter WASO and fewer long-wake episodes. The mechanism behind these associations is not clear. For postmenopausal women, starting HT use should be considered carefully in balance with other risks since the vascular side-effects of hormone replacement may exceed its beneficial effects on sleep.

## Background

Sleep in postmenopausal women is altered in ways which may have an adverse effect on health [1,2]. Explanations for sleep disturbances that occur during the menopausal transition and postmenopausal years include nocturnal hot flashes, mood disorders, and sleep disordered breathing [3-6]. Postmenopausal hormone therapy (HT) has been the standard treatment for many menopausal complaints, including hot flashes and insomnia [7-11]. However, given the large number of postmenopausal women experiencing sleep disturbance after the menopausal transition [12-14] and the consequences of poor sleep on daytime functioning, the relationship between HT and objectively measured sleep in older postmenopausal women needs further study.

Clinical and experimental studies offer compelling evidence that exogenous administration of sex hormones regulates many brain functions that are involved in the regulation of sleep [15-17]. Increased estrogen levels have been hypothesized to improve sleep by shortening sleep latencies [18]; reducing nocturnal restlessness and nighttime awakenings [11,19]; decreasing movement arousals [7]; improving sleep efficiency [20]; and increasing REM sleep [8,21,22]. Estrogen replacement has also been shown to reduce sleep disordered breathing in postmenopausal women [23,24] while exogenous administration of progestin produces sedative effects in both women and men [25,26]. Estrogen and progestin both improve vasomotor symptoms and may be beneficial for sleep disturbance [27,28]. Estrogen alone [7], but not estrogen plus progestin [29], alleviated the frequency of nocturnal movement arousals as measured by polysomnography. Given that data on the effects of estrogen on sleep quality (e.g. total sleep time, sleep efficiency, number of awakenings, wake after sleep onset, and sleep latency) in postmenopausal women are sparse, there is a clear need for contemporary research to clarify the inconsistency.

In this study, we examined data gathered in the Study of Osteoporotic Fractures (SOF), a longitudinal cohort study designed to examine the risk factors of osteoporotic fractures in women, to test the hypothesis that HT in postmenopausal women is associated with better sleep measured objectively by wrist actigraphy. This study population provides a unique opportunity to examine this question in a large community-dwelling cohort of postmenopausal women, with additional data collected to allow for adjustment of possible confounders.

## Methods

### Participants

The SOF is a longitudinal epidemiologic study of 10,366 women age 65 years or older, recruited from four study centers located in Baltimore, MD; Minneapolis, MN; Portland, OR; and the Monongahela Valley near Pittsburgh, PA. Women were excluded if they had a bilateral hip replacement or were unable to walk without assistance. The baseline SOF examinations were conducted from 1986-88, when 9,704 Caucasian women were recruited [30]. SOF was originally designed to investigate risk factors for osteoporosis and osteoporotic fractures, and initially African-American women were excluded from the study due to their low incidence of hip fractures, but from February 1997 to February 1998 662 African-American women were enrolled. At all subsequent visits, no exclusion criteria were used. All participants were community-dwelling at baseline. Since then follow-up exams have taken place approximately every two years.

The focus of this analysis was data gathered at the SOF year 16 or visit 8, which took place between January 2002 and February 2004. Of the 4,727 women at this visit, wrist actigraphy data were collected on 3,676 (78%) participants with clinic or home visits. Of the sample with wrist actigraphy, a total of 3,123 (85%) women had technically adequate actigraphy data and HT data available. Among these there were 424 current HT users, 1,289 past HT users, and 1,410 never HT users. The institutional review boards on human research approved the study at each institution, and all participating women provided written informed consent.

### Actigraphy

Participants wore the Sleep-watch-O (SleepWatch-O^®^, Ambulatory Monitoring, Inc) actigraph, a small, validated device worn on the wrist for highly effective sleep-wake inference from wrist activity [31,32]. Movement is measured by a piezoelectric linear accelerometer (sensitive to 0.003 g and above), which generates a voltage each time the actigraph is moved. These voltages are gathered continuously and summarized over 1-minute epochs. The actigraph collects movement data in 3 different modes; zero crossings mode (ZCM), time above threshold mode (TAT), and proportional integration mode (PIM). Data from the PIM mode was used in this analysis because it corresponded best to the gold standard of polysomnography (PSG) for estimation of sleep and wake in our population [33]. The actigraph was initialized in the clinic prior to the visit, and placed on the participant's nondominant wrist by the examiner during the visit. Women wore the actigraphs continuously for a minimum of three 24-hour periods (i.e., 72 hours). The average of the sleep parameters over all nights was used in this analysis to minimize interdaily variability. In general, scoring of actigraphy data was very reliable and highly predictive of total sleep time as measured by PSG [33].

ActionW-2 software (Ambulatory Monitoring, Inc.) was used to score the sleep and wake periods and compute sleep variables. All participants completed a daily sleep log which was used to edit the actigraphs data. The UCSD sleep scoring algorithm available in this software was used to differentiate sleep from wake for the PIM mode data. This algorithm calculates a moving average, which takes into account the activity levels immediately before and after the current minute to determine if the timepoint should be coded as sleep or wake. The following sleep parameters were calculated: Total sleep time (TST) as the mean minutes scored as nighttime sleep while in-bed; Sleep efficiency as the percentage of time (0-100%) the participant was sleeping while in-bed; Sleep latency as the number of minutes from the time the participant reported getting into bed to sleep onset as scored on the actigraph, defined as the when the participant achieved sleep for 20 continuous minutes after getting into bed; Wake after sleep onset (WASO) as the number of minutes scored as awake from sleep onset to the end of the last sleep episode while in-bed; Number of long-wake episodes defined as ≥ 5 minutes duration; Napping behavior was estimated as the total time while the participant was not in bed at night that was scored as sleeping by the sleep-scoring algorithm (this may include periods of extreme inactivity).

Sleep parameters were analyzed both as continuous measures and as categorical exposure variables based on current beliefs about clinically relevant values of sleep parameters in older adults. Total sleep time was categorized as <6 hours, 6-8 hours and >8 hours. Sleep efficiency was categorized as <70% and ≥ 70%. Sleep latency was categorized as <60 minutes and ≥ 60 minutes. WASO was categorized as <90 minutes and ≥ 90 minutes. Number of long-wake episodes was categorized as <8 and ≥ 8. Napping was categorized as <2 hours and ≥ 2 hours.

### Postmenopausal Hormone Use

Participants were asked about HT at every follow-up visit approximately every two years for 16 years up to this visit. Women were divided into three HT groups: never users; past users (stopped use prior to Visit 8); current users (used at Visit 8). Long versus short term current HT was examined by analyzing associations for participants using HT for ≤ 5 (n = 86) and >5 years (n = 338). Among past HT users, associations among those stopping ≤ 5 (n = 454) and >5 (n = 835) years ago were examined.

### Other Measurements

All participants completed questionnaire data, which included questions about medical history, self-reported health, smoking status, alcohol use, marital status, and whether or not the participant walked for exercise. The Geriatric Depression Scale (GDS) was used to assess depressive symptoms with the standard cutoff of ≥ 6 symptoms used to define depression. Anxiety was assessed using the Goldberg Anxiety Scale. During the home or clinic visits current medication use within the last 2 weeks was assessed by examination of medications, and a computerized medication coding dictionary was used to categorize these medications. The Mini-Mental State Examination (MMSE) was administered to assess cognitive function, with higher scores on a scale of 0 to 30 representing better cognition. Physical activity was assessed by asking participants how many blocks they normally walked for exercise per day. Body weight and height were measured, and body mass index (BMI) was calculated as Kg/m^2^.

### Statistical analysis

Characteristics known to be related to HT were summarized using means and SDs for continuous data and percentages for categorical data. To identify potential confounders, we considered a list of predictors thought to be associated with HT and sleep parameters based on biological plausibility or previous studies including age, clinic site, race, BMI, anxiety, depression, cognitive function, alcohol use, smoking status, education, marital status, self-reported health status, physical activity, antidepressant use, prior hysterectomy, and medical conditions (cardiovascular disease, cancer, hypertension and diabetes). We compared characteristics between current, past and never HT users using 3-way ANOVA for continuous covariates and chi square tests for categorical data. All assumptions of the ANOVA model were tested and p-values were calculated using the Wilcoxon non-parametric test for skewed covariates. To assess potential confounding we performed univariate tests to determine the association between the each of the sleep variables and the covariates. Variables that were significantly related (p < 0.10) to at least one sleep outcome measure and HT were included in the final multivariate analyses. To determine the relationship between HT and continuous measures of sleep parameters in the multivariate analysis, we compared the adjusted means across the 3 categories using ANOVA pairwise tests from generalized linear models. Analyses were performed in which propensity scores were calculated indicating the likelihood of HT use based on a ordinal logistic regression model with HT as the outcome adjusted for the covariates in the multivariate model [34]. This propensity score was then used in place of the covariates in the models. Because use of propensity scores did not substantially alter the findings regarding the association between HT and likelihood of sleep disturbances, models adjusted for multiple covariates are presented in this article. Multinomial logistic regression models were also used to examine the relationship between HT and categorized sleep parameters. We modeled the odds of sleep disturbance for the following categories: sleep efficiency <70% compared with ≥ 70%; sleep latency ≥ 60 minutes compared with <60 minutes and; WASO ≥ 90 minutes compared with <90 minutes; number of long-wake episodes ≥ 8 compared with <8; and napping ≥ 2 hours compared with <2 hours. Polytomous logistic regression was used to simultaneously estimate the odds of total sleep time <6 hours and >8 hours compared with 6-8 hours. Statistical analysis was performed using the statistical software program SAS version 9.1 (SAS Institute, Inc., Cary, NC).

## Results

### Characteristics of the study population

The analysis cohort for this study was composed of 3,123 women (mean age 84 ± 4 years, range 77-99 years) with actigraphy measurements. We previously compared the overall distribution of subject characteristics and health conditions of the 3,123 women with wrist actigraphy to the 1,795 who did not have actigraphy measured [35]. The latter group was on average 1.4 years older, had higher rates of IADL impairment and stroke, had a worse mean depression score, lower cognitive functioning, and were more likely to be diagnosed with Alzheimer's disease. This group also and experienced an almost 2-fold increased mortality rate compared to women with actigraphy measurements.

Among the 3,123 women with wrist actigraphy, there were 424 current HT users, 1,289 past HT users, and 1,410 never HT users (Table 1). Current HT users were slightly younger and more likely to be married, have a higher education, exercise more, have better cognitive function, have had a hysterectomy when compared to both other groups. Never HT users were more likely to be African American, have a higher BMI and more medical conditions when compared to both other groups. Past HT users were more likely to have higher anxiety scale sores when compared to both other groups. Both current and past HT users were more likely to be diagnosed with depression when compared to never users. The majority of current HT users were taking conjugated estrogens (88%) including Premarin (62%) and Prempro (5%). Approximately 15% of current HT users were taking estrogen and progestin.

**Table 1:** Visit 8 characteristics among postmenopausal hormone therapy (HT) categories from the SOF Cohort.

Characteristics	Current	Past	Never	
	(N = 424)	(N = 1,289)	(N = 1,410)	P-value*
Age (y), mean +/- SD	82.9 +/- 3.4	83.8 +/- 3.4	83.6 +/- 4.2	0.0002
Body mass index (kg/m^2^), mean +/- SD	26.2 +/- 4.6	26.6 +/- 4.7	27.7 +/- 5.3	<.0001
Married, n (%)	149 (35.2)	378 (29.4)	299 (21.2)	<.0001
African-American, n (%)	34 (8.0)	29 (2.3)	265 (18.8)	<.0001
Clinic site, n +/- SD				<.0001
BALTIMORE	70 (16.5)	238 (18.5)	240 (17.0)	
MINNEAPOLIS	131 (30.9)	464 (36.0)	422 (29.9)	<.0001
PITTSBURGH	49 (11.6)	280 (21.7)	537 (38.1)	<.0001
PORTLAND	174 (41.0)	307 (23.8)	211 (15.0)	<.0001
Education (y), mean +/- SD	13.3 +/- 2.6	13.1 +/- 2.6	12.4 +/- 2.8	<.0001
Age at menopause, mean +/- SD	47.7 +/- 6.4	48.5 +/- 5.6	47.9 +/- 5.9	0.0181
Years of menopause, mean +/- SD	34.6 +/- 6.9	35.2 +/- 6.5	34.6 +/- 7.6	0.3451
Any alcohol intake in the past 30 days, n (%)	188 (44.4)	583 (45.3)	514 (36.5)	<.0001
Current smoker, n (%)	10 (2.4)	32 (2.5)	43 (3.1)	0.593
Self-reported health status, n (%)				0.4438
Poor/very poor	7 (1.7)	28 (2.2)	36 (2.6)	
Fair	88 (20.8)	275 (21.4)	330 (23.4)	0.4438
Good/excellent	328 (77.5)	984 (76.5)	1044 (74.0)	0.4438
Medical condition (0-4)	1.2 +/- 0.9	1.3 +/- 1.0	1.3 +/- 0.9	0.1621
Goldberg Anxiety Scale score, mean +/- SD	1.3 +/- 2.2	1.5 +/- 2.3	1.2 +/- 2.1	0.0007
Geriatric Depression Scale score, mean +/- SD	2.2 +/- 2.3	2.5 +/- 2.6	2.4 +/- 2.7	0.0157
Diagnosed with depression	124 (8.81)	185 (14.35)	64 (15.13)	<.0001
MMSE score, mean +/- SD	28.1 +/- 1.7	28.0 +/- 2.0	27.7 +/- 2.1	<.0001
Walks for exercise (yes), n (%)	182 (43.2)	487 (38.3)	479 (34.4)	0.0028
Blocks walked for exercise (daily), mean +/- SD	9.8 +/- 12.2	8.14 +/- 9.9	7.5 +/- 9.7	0.0072
Currently taking antidepressants, n (%)	43 (10.1)	119 (9.2)	104 (7.4)	0.0984
Current sleep medication user, n (%)	1 (0.2)	26 (2.0)	8 (0.6)	0.0003
Prior hysterectomy, n (%)	285 (67.4)	551 (42.9)	409 (29.0)	<.0001

* Values of p for continuous data are from a t test for normally distributed data and a Wilcoxon rank sum test for skewed data. Values of p for categorical data are from a chi-squared test test for homogeneity.

SOF = Study of Osteoporotic Fractures; MMSE = Mini-Mental State Examination; SD = standard deviation.

### HT and sleep

Women currently taking HT compared to never users were less likely to have their sleep interrupted by waking after falling asleep (Table 2), as indicated by fewer long-wake (≥ 5 minutes) episodes (6.5 vs. 7.1, P = 0.004) and less WASO time (76 vs. 82 minutes, P = 0.03) after adjustment for multiple potential confounders (age, clinic site, race, BMI, anxiety, depression, cognitive function, alcohol use, education, marital status, self-reported health status, number of blocks walked for exercise per day, prior hysterectomy and medical conditions). Sleep latency did not differ across the groups, nor did sleep efficiency or napping. Past HT users had significantly more total sleep time than never users (413 vs. 403 minutes, P = 0.002). Among current HT users, length of usage (≤ 5 and >5 years) and among past HT users, time since stopping did not influence group differences in sleep indicators (data not shown).

**Table 2:** Associations between postmenopausal hormone therapy (HT) and sleep parameters.

	HT use		
	
Parameter	Current	Past	Never
	(N = 424)	(N = 1,289)	(N = 1,410)
Total sleep time, minutes (SD)	407 (5)	413 (4)	403 (4)^a^
Sleep efficiency, % (SD)	77.3 (0.8)	76.9 (0.7)	76.1 (0.6)
Sleep latency, minutes (SD)	41.4 (2.7)	43.5 (2.2)	44.7 (2.2)
Wake after sleep onset, minutes (SD)	76.0 (3.3)	79.1 (2.7)	82.1 (2.7)^b^
Long wake episodes, # (SD)	6.5 (0.2)	6.9 (0.2)^b^	7.1 (0.2)^c^
Napping, minutes (SD)	77.1 (4.5)	80.0 (3.7)	80.5 (3.6)

a P < 0.01 vs. Past HT use

b P < 0.05 vs. Current HT use

c P < 0.01 vs. Current HT use

Adjusted for age, clinic site, race, BMI, cognitive function, number of blocks walked for exercise per day, depression, anxiety, education, marital status, age at menopause, alcohol use, prior hysterectomy and medical conditions.

Compared to current HT users, women who never used HT had significantly higher odds of ≥ 8 long-wake episodes (odds ratio (OR), 1.58; 95% confidence interval (CI), 1.18-2.12; p = 0.002) and wake after sleep onset ≥ 90 minutes (OR, 1.37; 95% CI, 1.02-1.83; p = 0.03) (Table 3). Not statistically significant were higher odds of low sleep efficiency <70% (OR, 1.37; 95% CI, 0.98-1.92) and napping ≥ 2 hours (OR, 1.35; 95% CI, 0.94-1.92) (Table 3). Odds of having lower total sleep time or higher sleep latency were not different across the groups. There was no effect of progestin use or duration of HT use on the results and results did not differ when estrogen+progestin users were excluded from the analysis.

**Table 3:** Associations between postmenopausal hormone therapy (HT) and categorized sleep parameters.

		HT use		
		
Parameter		Current	Past	Never
		(N = 424)	(N = 1,289)	(N = 1,410)
Total sleep time	<6 hours^a^	1.00 (Ref.)	0.99 (0.85 - 1.15)	1.14 (0.85 - 1.53)
	>8 hours^a^	1.00 (Ref.)	1.17 (0.97 - 1.40)	1.02 (0.69 - 1.51)
Sleep efficiency	<70%^b^	1.00 (Ref.)	1.03 (0.87 - 1.21)	1.37 (0.98 - 1.92)
Sleep latency	>= 60 minutes^c^	1.00 (Ref.)	1.06 (0.90 - 1.25)	1.15 (0.82 - 1.60)
Wake after sleep onset	>= 90 minutes^d^	1.00 (Ref.)	1.03 (0.90 - 1.19)	1.37 (1.02 - 1.83)
Long wake episodes	>= 8^e^	1.00 (Ref.)	1.10 (0.95 - 1.27)	1.58 (1.18 - 2.12)
Napping	>= 2 hours^f^	1.00 (Ref.)	1.13 (0.95 - 1.34)	1.35 (0.94 - 1.92)

a Compared with 6-8 hours

b Compared with ≥ 70%

c Compared with <60 minutes

d Compared with <90 minutes

e Compared with <8 episodes

f Compared with <2 hours

Adjusted for age, clinic site, race, BMI, cognitive function, number of blocks walked for exercise per day, depression, anxiety, education, marital status, age at menopause, alcohol use, prior hysterectomy and medical conditions.

## Discussion

To our knowledge, this is the first study in older, community-dwelling postmenopausal women that has evaluated the relationship between long-term HT and objectively measured sleep. These results are consistent with findings from a large number of epidemiological studies that support a relationship between HT and improved sleep characteristics [1,2]. A limited number of studies including menopausal women not reporting vasomotor symptoms have demonstrated that HT use relieved insomnia [11] and reduced sleep disturbance [27], suggesting a mechanism independent of hot flashes. A placebo-controlled study of HT in postmenopausal women reported decreased wakefulness after sleep onset, and increased total sleep time after the initiation of HT and enhanced REM sleep [21]. Another comparison of placebo with HT showed a reduction in the number of sleep-disordered breathing episodes and a decreased duration of hypopneas with HT [24].

Sleep difficulties in older postmenopausal women may be influenced by the emergence of coexisting medical conditions and the presence of other hormonal, physiologic, and even psychosocial factors [36-38]. Estrogen regulates the synthesis and release of neurotransmitters and neuromodulators that affect many brain functions including mood, behavior, cognition and sleep [15-17,21,39,40]. For example, the serotonergic system is regulated by ovarian steroids and by estrogen and progesterone treatment [39]. In addition, sex hormone receptors are present in the suprachiasmatic nucleus (SCN) [41], the key biologic clock in the brain that orchestrates circadian biological rhythms, such as the rhythms of hormones, body temperature, sleep and mood. These experimental findings may provide a potential neuroendocrine mechanism by which HT can act to improve SCN-related rhythm disturbances, such as body temperature, mood and sleep [41].

A small number of observational studies [5,42,43] have examined the role of HT in preventing or alleviating sleep-disordered breathing (SDB), which is more common among postmenopausal women and may be a confounder in the present analysis. This is also an important consideration since wrist actigraphy may underestimate arousal/fragmentation index and WASO as measured by PSG in severe obstructive sleep apnea patients [44]. One small study [42] found that postmenopausal women taking HT had a lower prevalence of SDB compared with postmenopausal women not taking HT (based on fewer than 30 women with SDB). The presence of SDB in this study appeared to be associated exclusively with obesity and controlling for age and BMI attenuated the association between HT and SDB [42]. A second study [43] found that HT users had lower odds of SDB but that controlling for BMI removed the association between menopause and SDB. A third study [5] identified a reduced risk of SDB among HT users and that the association remained significant after adjustment for determinants of SDB. These data combined suggest that menopause is a significant risk factor for SDB in women and that HT appears to be associated with reduced risk. However, it should be noted that age and BMI are significant risk factors for SDB and that the inverse relationship between HT and SDB was weakest among the oldest age group ≥ 70 years [5,42], which is similar to the age group in the current study.

Several methodologic and etiologic factors may be contributing to the inconsistent associations previously reported for HT and sleep, including differences in study population, hormone formulation and dose, and duration of study [7,8,21,29]. In this study, current HT users had a higher education, exercised more, had fewer medical conditions and better cognitive function but were also more likely to be diagnosed with depression compared to past and never postmenopausal hormone users. Multivariate adjustment suggests that the association of HT with improved sleep is independent of these factors. However, it is possible that HT may have no direct causal association with sleep improvement and may be influencing other age-related factors that are benefiting sleep but are not accounted for in our analysis. We found no sleep quality differences between those participants currently using HT for ≤ 5 and >5 years or between past HT users who stopped ≤ 5 and >5 years prior to this study. HT formulations are not uniform and consist of various estrogen and progestin combinations and doses that may lead to differential effects on sleep. In this study, results did not differ when estrogen and progestin users were excluded from the analysis. However, as there were fewer than 80 progestin users among the SOF participants with wrist actigraphy, progestin use could still be a potential confounder of this relationship.

Our study had a number of strengths, including a large cohort of community-dwelling postmenopausal women with no inclusion requirements regarding sleep disorders. Several different sleep parameters were examined, all of which were gathered objectively, and there were extensive data for adjustment of possible confounders. To avoid any misclassifications of HT usage prior to actigraphy, measurements of HT usage were collected approximately every two years for 16 years. For this analysis, it is likely that hot flashes were uncommon in the study participants who were 77-99 years at the sleep exam. This analysis also had several limitations. We are unable determine causality since actigraphy was collected at a single time and we cannot rule out that previous sleep problems influenced HT usage. Findings are for older women, with a mean age of 84 ± 4 years (range 77-99 years) and may not be generalizable to younger women undergoing menopause. We adjusted for education as a surrogate for SES but this may be an inadequate measure of SES in this cohort of elderly women. The individuals who had actigraphy were somewhat healthier than those who did not have these measures, and thus, it is possible that different associations may have been observed with inclusion of these women. The lack of PSG limits our ability to account for sleep apnea in these analyses. Lastly, confounding by unknown or other unmeasured confounding factors could have resulted in biased estimates of effect.

## Conclusions

Our findings suggest that postmenopausal women currently using HT had improved sleep quality for two out of five objective measurements. Both measures, shorter WASO and fewer long-wake episodes, are specifically related to sleep fragmentation. The mechanism behind this association is not clear but multivariate adjustment suggests that the beneficial effects of HT are not explained by other behavioral, aging- and health-related variables. While the majority of women start HT to relieve menopausal symptoms, some continue to use HT beyond the menopausal transition. In this study 14% of participants were using HT at the 16^th ^year of the SOF (mean age of 83 ± 4 years). For an individual postmenopausal woman, however, starting HT use should be considered carefully in balance with other risks since the vascular side-effects of hormone replacement may exceed its beneficial effects on sleep [45-47].

## Competing interests

The authors declare that they have no competing interests.

## Authors' contributions

GT conceived of the project, participated in the design of the study and drafted the manuscript. NP, TB performed statistical analyses and helped to draft the manuscript. MP participated in the design of the study and helped to draft the manuscript. SA-I, KE, JC, SR, NL, TH, KY, SC, KS conceived of the study, participated in study design and coordination and helped to draft the manuscript

All authors read and approved the final manuscript.

## Pre-publication history

The pre-publication history for this paper can be accessed here:

http://www.biomedcentral.com/1472-6874/10/15/prepub
